# Mitochondrial Utilization of Competing Fuels Is Altered in Insulin Resistant Skeletal Muscle of Non-obese Rats (Goto-Kakizaki)

**DOI:** 10.3389/fphys.2020.00677

**Published:** 2020-06-16

**Authors:** Nicola Lai, Ciarán E. Fealy, Chinna M. Kummitha, Silvia Cabras, John P. Kirwan, Charles L. Hoppel

**Affiliations:** ^1^Department of Electrical and Computer Engineering, Old Dominion University, Norfolk, VA, United States; ^2^Biomedical Engineering Institute, Old Dominion University, Norfolk, VA, United States; ^3^Department of Mechanical, Chemical and Materials Engineering, University of Cagliari, Cagliari, Italy; ^4^Department of Biomedical Engineering, Case Western Reserve University, Cleveland, OH, United States; ^5^Center for Mitochondrial Disease, Case Western Reserve University, Cleveland, OH, United States; ^6^Department of Pathobiology, Lerner Research Institute, Cleveland Clinic, Cleveland, OH, United States; ^7^Department of Physiology and Biophysics, Case Western Reserve University, Cleveland, OH, United States; ^8^Pennington Biomedical Research Center, Baton Rouge, LA, United States; ^9^Department of Pharmacology, Case Western Reserve University, Cleveland, OH, United States; ^10^Department of Medicine, School of Medicine, Case Western Reserve University, Cleveland, OH, United States

**Keywords:** metabolic flexibility, fatty acid oxidation, bioenergetic, oxidative phosphorylation, diabetes

## Abstract

**Aim:**

Insulin-resistant skeletal muscle is characterized by metabolic inflexibility with associated alterations in substrate selection, mediated by peroxisome-proliferator activated receptor δ (PPARδ). Although it is established that PPARδ contributes to the alteration of energy metabolism, it is not clear whether it plays a role in mitochondrial fuel competition. While nutrient overload may impair metabolic flexibility by fuel congestion within mitochondria, in absence of obesity defects at a mitochondrial level have not yet been excluded. We sought to determine whether reduced PPARδ content in insulin-resistant rat skeletal muscle of a non-obese rat model of T2DM (Goto-Kakizaki, GK) ameliorate the inhibitory effect of fatty acid (i.e., palmitoylcarnitine) on mitochondrial carbohydrate oxidization (i.e., pyruvate) in muscle fibers.

**Methods:**

Bioenergetic function was characterized in oxidative soleus (S) and glycolytic white gastrocnemius (WG) muscles with measurement of respiration rates in permeabilized fibers in the presence of complex I, II, IV, and fatty acid substrates. Mitochondrial content was measured by citrate synthase (CS) and succinate dehydrogenase activity (SDH). Western blot was used to determine protein expression of PPARδ, PDK isoform 2 and 4.

**Results:**

CS and SDH activity, key markers of mitochondrial content, were reduced by ∼10–30% in diabetic vs. control, and the effect was evident in both oxidative and glycolytic muscles. PPARδ (*p* < 0.01), PDK2 (*p* < 0.01), and PDK4 (*p* = 0.06) protein content was reduced in GK animals compared to Wistar rats (*N* = 6 per group). *Ex vivo* respiration rates in permeabilized muscle fibers determined in the presence of complex I, II, IV, and fatty acid substrates, suggested unaltered mitochondrial bioenergetic function in T2DM muscle. Respiration in the presence of pyruvate was higher compared to palmitoylcarnitine in both animal groups and fiber types. Moreover, respiration rates in the presence of both palmitoylcarnitine and pyruvate were reduced by 25 ± 6% (S), 37 ± 6% (WG) and 63 ± 6% (S), 57 ± 8% (WG) compared to pyruvate for both controls and GK, respectively. The inhibitory effect of palmitoylcarnitine on respiration was significantly greater in GK than controls (*p* < 10^–3^).

**Conclusion:**

With competing fuels, the presence of fatty acids diminishes mitochondria ability to utilize carbohydrate derived substrates in insulin-resistant muscle despite reduced PPARδ content.

## Introduction

Skeletal muscle insulin resistance is a primary defect in the pathogenesis of type 2 diabetes mellitus (T2DM). Although the mechanism that underlies the development of insulin resistance is not fully understood, a large body of research implicates excessive accumulation of intramyocellular lipids (IMCL) and impaired mitochondrial function (Shulman, 2004; Abdul-Ghani and Defronzo, 2010). In contrast, other studies indicate that these conditions are not strictly required to induce insulin resistance (Wicks et al., 2015; Vandanmagsar et al., 2016). The loss of metabolic flexibility, defined as the capacity to adapt fuel (carbohydrate and fat) utilization to fuel availability (Kelley and Mandarino, 2000), is a hallmark of insulin resistance and T2DM. It has been suggested that nutrient overload may impair metabolic flexibility due to fuel congestion within mitochondria (Muoio, 2014), however, defects at a mitochondrial level have not yet been excluded. Mitochondrial fuel competition experiments can help to clarify whether metabolic inflexibility factors are present within mitochondria. Thus, an altered response to fuel competition in healthy vs. diabetic muscle mitochondria, in the absence of nutrient overload, could indicate the presence of intrinsic factors influencing metabolic flexibility.

The strategy of stimulating mitochondrial respiration with competing substrates is used to evaluate substrate utilization in conditions mimicking the transition from fasting to the fed state (Muoio et al., 2012; Muoio, 2014). Fuel competition studies show that mitochondria isolated from healthy skeletal muscle of mouse, rat, and humans, preferentially utilize free fatty acid metabolites over carbohydrate substrates for ATP synthesis and respiration (Abdul-Ghani et al., 2008; Kuzmiak-Glancy and Willis, 2014). This evidence suggests that metabolic impairment in utilizing competing substrates may be present within mitochondria where transcription factors can have a role. A computational study based on metabolic control analysis (Cortassa et al., 2019) suggested that cytoplasmic and mitochondrial metabolic networks are responsible for the regulation of fatty acid and glucose utilization.

Peroxisome proliferator-activated receptors (PPAR) δ/β (hereafter PPARδ) (Ehrenborg and Krook, 2009; Phua et al., 2018) which is reduced in T2DM (Mensink et al., 2007; Shen et al., 2008), regulates skeletal muscle substrate utilization at the transcriptional level. PPARδ is reported to induce upregulation of (pyruvate dehydrogenase kinase) PDK isoform 2 (PDK2) and 4 (PDK4) (Constantin et al., 2007), which regulate the activity of the pyruvate dehydrogenase (PDH) complex, while also modifying genes responsible for fatty acid transport, b-oxidation (Tanaka et al., 2003; Wang et al., 2003, 2004; Phua et al., 2018), and mitochondrial biogenesis, via peroxisome proliferator-activated gamma coactivator 1-alpha (PGC1-α) (Schuler et al., 2006; Phua et al., 2018). Previous studies using transgenic mice or PPARδ activators have established that PPARδ contributes to skeletal muscle fuel selection (Ehrenborg and Krook, 2009) without any effects on mitochondrial function (Constantin et al., 2007). Nevertheless, it is unclear whether reduced PPARδ expression, as in T2DM, alters fuel competition during mitochondrial substrate utilization. Furthermore, fuel competition has not been studied in mitochondria of insulin resistant skeletal muscle. To determine whether free fatty acid metabolites reduce mitochondrial utilization of carbohydrate substrates in insulin resistant skeletal muscle, as observed in healthy skeletal muscle mitochondria, requires the assessment of fuel competition in mitochondria of T2DM in the absence of nutrient oversupply as seen in healthy muscle. The presence of obesity has been reported to cause metabolic inflexibility (Ukropcova et al., 2005; Aucouturier et al., 2011; Muoio, 2014), thus, by using a non-obese model of T2DM we were able to minimize the potential confounding effect of obesity-related metabolic disturbance on mitochondrial fuel utilization.

The Goto-Kakizaki (GK) rat is a widely used lean model of T2DM. Studies have shown that skeletal muscle in the GK rat is insulin resistant even in the absence of obesity (Goto and Kakizaki, 1981; Steiler et al., 2003), displays preserved mitochondrial function (Lai et al., 2017) and has reduced PPARδ gene expression (Shen et al., 2008) with concomitant hyperglycemia and hyperinsulinemia (Steiler et al., 2003; Kuwabara et al., 2017; Lai et al., 2017), despite normal levels of plasma non-esterified fatty acids (NEFA), and normal skeletal muscle glycogen and IMCL content (Macia et al., 2015). Thus, we elected to use this animal model to study mitochondrial fuel competition in insulin resistant skeletal muscle that lacks obesity-associated nutrient oversupply (Muoio, 2014).

Moreover, the competing effect of fatty acids on carbohydrate substrate utilization may differ in mitochondria of red oxidative and white glycolytic muscle fibers, given their differing ability to oxidize fat (Pande and Blanchaer, 1971; Carroll et al., 1983; Jackman and Willis, 1996; Glancy and Balaban, 2011; Li et al., 2012). The capacity of glycolytic muscle fibers to utilize fat is less than oxidative muscle fibers whereas the effect of fatty acids on glucose utilization in oxidative differed from that in glycolytic fibers (Jenkins et al., 1988). Furthermore, it is not known whether the different biochemical properties of oxidative and glycolytic fibers affect substrate selection in the presence of competing fuels.

We proposed that reduced PPARδ in insulin-resistant skeletal muscle results in impaired mitochondrial fat oxidation by altering substrate selection pathways and promoting greater responsiveness to carbohydrate derived substrate. To test this hypothesis, we measured skeletal muscle PPARδ and PDK4/2 protein content, muscle fiber respiration, and fuel competition between pyruvate and palmitoylcarnitine as representative substrates for carbohydrate and fat oxidation in oxidative soleus and glycolytic white gastrocnemius from GK rats compared to lean healthy controls.

## Results

### Animal Model

The animal characteristics are reported in Table 1. The animals used in this study are the same of those used in our previous study (Lai et al., 2017, 2020). The diabetic (GK) rats had a significant lower body weight than control (W) rats at both age groups. While the body weight of W rats significantly increased by 24% from 18 to 28 weeks that of GK rats did not change. The GK rats were hyperinsulinemic and hyperglycemic at both age groups.

**TABLE 1:** Animal characteristics: body weight, insulin, and glucose concentration in blood.

	Unit	Wistar	GK
Body weight	(g)	590 ± 58	389 ± 21*
Insulin	(ng mL^–1^)	2.4 ± 2.1	5.1 ± 1.4*
Glucose	(mM)	6.5 ± 1.7	17.4 ± 3*

*(P < 0.03) Wistar 28 weeks vs. GK-28 week (n = 6).

### Mitochondrial Content

Citrate synthase (CS) and succinate dehydrogenase (SDH) activities were used to assess mitochondrial content in soleus (S) and white gastrocnemius (WG) muscles (Table 2). In GK rats, CS activity was 10 ± 3% lower in the S (*p* < 0.01) and 15 ± 4% lower in the WG muscle (*p* < 0.04) compared to CON. The SDH activity was 27 ± 4% lower (*p* < 0.01) in both muscle groups in the GK animals compared to Wistar.

**TABLE 2:** Specific activities of mitochondrial marker enzymes (CS, SDH, U g^–1^) in white gastrocnemius (WG) and soleus (S) of GK and Control rats.

		Wistar	GK
CS	WG	16.8 ± 0.6	14 ± 0.6*
	S	33.3 ± 0.6^§^	29.2 ± 0.8^*,§^
SDH	WG	0.42 ± 0.03	0.3 ± 0.02*
	S	1.8 ± 0.04^§^	1.3 ± 0.05^*,§^

*p < 10^–2^ Wistar vs. GK, ^§^ p < 10^–4^ S vs. WG.

### Respiration Rates in Oxidative and Glycolytic Fibers

**FIGURE 1:**
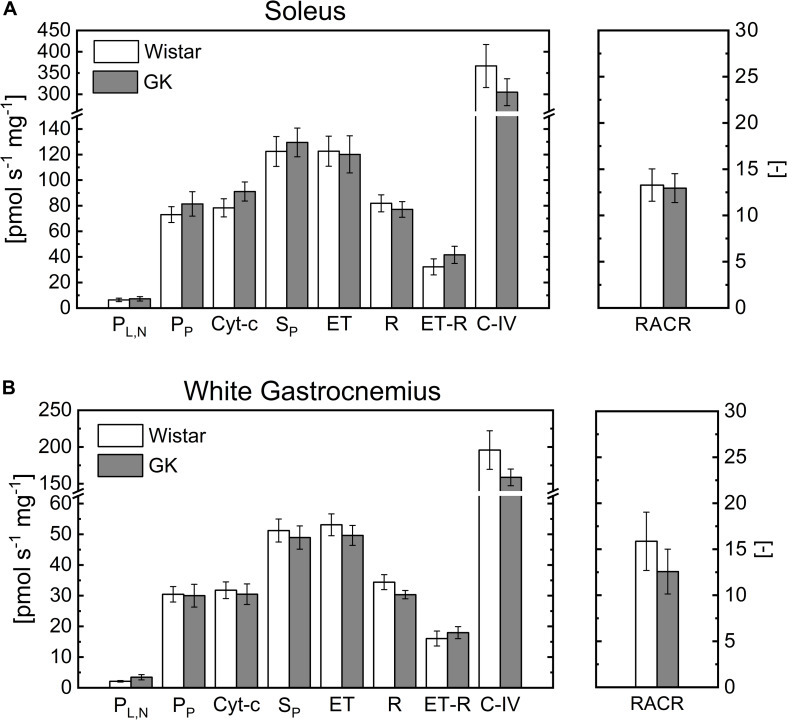
Respiration rates in permeabilized soleus **(A)** and white gastrocnemius **(B)** muscles of control and GK rats obtained under leak state (P_L,N_) and oxphos state (P_P_) with malate and pyruvate, cytochrome c (Cyt-c), Succinate (S_P_), uncoupled mitochondria to measure maximal electron transport chain capacity (ET), rotenone (R). Also, ET-R indicates the respiration rate difference between that indicated with ET and R; CIV indicates the respiration rate difference between that with ascorbate plus TMPD and azide; Respiratory acceptor control ratio (RACR). Data are mean ± SEM (*n* = 6). For soleus, the respiration rate obtained with cytochromes c is 8 ± 3% and 14 ± 7% greater than that in presence of only P for control and GK, respectively. And for white gastrocnemius is 5 ± 4% and 2 ± 2%, respectively.

Respiration rates in permeabilized S and WG fibers were similar for both experimental groups (Figure 1). The basal respiration rate determined in the presence of malate and pyruvate and absence of ADP in both S (Figure 1A) and WG (Figure 1B) permeabilized fibers (leak state with no adenylates (Gnaiger et al., 2019), P_L,N_) was similar for GK and Wistar animals. Oxphos state (i.e., State 3) respiration rate in the presence of pyruvate (P_P_) and succinate (S_P_), which supply electrons to complex I and II respectively, was not different between GK and Wistar for both S and WG fibers (Figure 1). Uncoupled mitochondrial respiration rate (ET) was similar to OXPHOS determined with coupled mitochondria (S_P_), indicating that the phosphorylation system is not limiting OXPHOS (Figure 1). After inhibiting complex III with antimycin A, ascorbate and TMPD were added to measure uncoupled complex IV respiration rate. As observed for other substrates, complex IV respiration rate was unaltered in GK (Figure 1).

CS and SDH activities were lower in GK vs. control rats for both muscle fibers. Thus, respiration rates were normalized to CS and SDH activity. This correction resulted in higher respiration rates in GK than control, however, this increase was not statistically significant for the rates normalized to CS (Supplementary Figure 1). When the rates were normalized to SDH activity, those obtained with pyruvate and succinate were greater in GK than control rats only for S fibers (Supplementary Figure 2A, *p* < 0.03).

### Competing Substrate Utilization in Mitochondrial Metabolism

**FIGURE 2:**
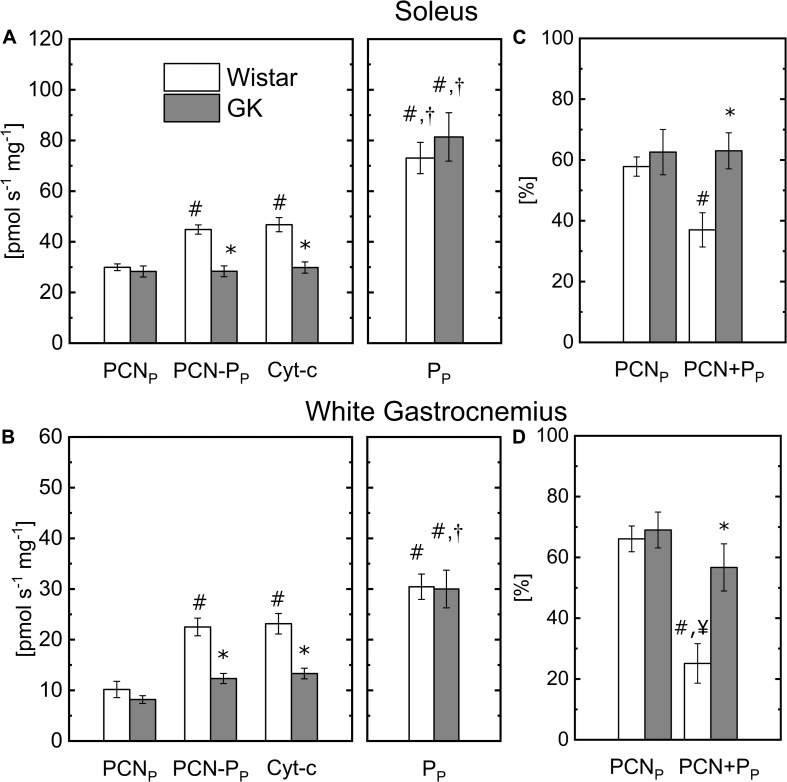
Comparison of the respiration rates obtained with malate plus palmitoylcarnitine (PCN_P_), or pyruvate (P_P_) or PCN + P_P_ or PCN + P_P_ with cytochromes c (Cyt-c) in permeabilized soleus **(A)** and white gastrocnemius **(B)** fibers of control and GK rats. For soleus, the respiration rate obtained with cytochromes c is 4 ± 2% and 1 ± 1% greater than that in presence of PCN + P_P_ for control and GK, respectively. And for white gastrocnemius it is 3 ± 2% and 8 ± 4%, respectively. Comparison of the respiration rate with PCN_P_ and PCN + P_P_ changes relative to that obtained with P_P_ for soleus **(C)** and white gastrocnemius **(D)** fibers of control and GK rats; Influence of substrate within group for soleus and white gastrocnemius muscles (*p* < 10^– 5^): (^#^) Statistically different from PCN_P_, (^†^) Statistically different from PCN + P_P_. Influence of insulin resistance in presence of PCN + P_P_ substrates for soleus and white gastrocnemius muscles (*p* < 10^–3^): (*) Statistically different from control soleus and white gastrocnemius. Data are mean ± SEM (*n* = 6). (^¥^) Statistically different from GK PCN_P_. Data are mean ± SEM (*n* = 6).

To determine the capacity of permeabilized muscle fibers to metabolize fatty acids, palmitoylcarnitine (PCN) was used as a substrate to supply mitochondrial b-oxidation. The Oxphos state respiration rate state (PCN_P_) in presence of malate + PCN was similar in Wistar and GK for both muscle fibers (Figure 2). Oxphos state respiration rate (P_P_) obtained in the presence of pyruvate (Figure 1) also is reported in Figure 2 to facilitate a comparison with the respiration rates obtained with PCN or PCN and pyruvate (PCN + P_P_). In the Wistar group, the addition of pyruvate in the presence of PCN significantly increased mitochondrial respiration rates from 30 ± 4 (PCN_P_) to 44.8 ± 1.8 (PCN + P_P_) pmol s^–1^ mg^–1^ ww in permeabilized fibers from S (Figure 2A), and 10 ± 1.6 (PCN_P_) to 22.5 ± 1.7 (PCN + P_P_) pmol s^–1^ mg^–1^ ww for WG fibers (Figure 2B). In the GK group, the addition of pyruvate did not affect mitochondrial respiration rate in S fibers (28 ± 2.2 with PCN_P_ and 28.4 ± 2.1 with PCN + P_P_ pmol s^–1^ mg^–1^, Figure 2A), but increased respiration rates from 8.2 ± 0.7 (PCN_P_) to 12.3 ± 1.0 (PCN + P_P_) pmol s^–1^ mg^–1^ ww in WG (Figure 2B). The decrease of the respiration rate with P in presence of PCN was greater in GK than control for both muscle fiber types (Figures 2C,D). In both muscle groups, mitochondrial respiration rate determined in the presence of both PCN and pyruvate was significantly lower in the GK rats compared to Wistar (Figure 2). In the Wistar group, the respiration rate observed with PCN and P was reduced by 37 ± 6% and 25 ± 6% of that determined with pyruvate in S and WG muscle fibers, respectively. In GK, the respiration rate observed with PCN and P was reduced by approximately 63 ± 6% and 57 ± 8% of that determined with pyruvate in S and WG muscle fibers, respectively.

To determine mitochondrial membrane integrity, mitochondrial respiration rate was measured with and without cytochrome c in the presence of complex I substrate (malate + pyruvate) (Figure 1) or in the presence of PCN + P_P_ (Figure 2), with a saturating concentration of ADP. Addition of cytochrome c did not significantly (*p* > 0.6) affect the respiration rates for both sets of experiments (Figures 1, 2). The increase in respiration rate in the presence of cytochrome c is reported in the figure legends for both muscle fiber types.

### Protein Expression

**FIGURE 3:**
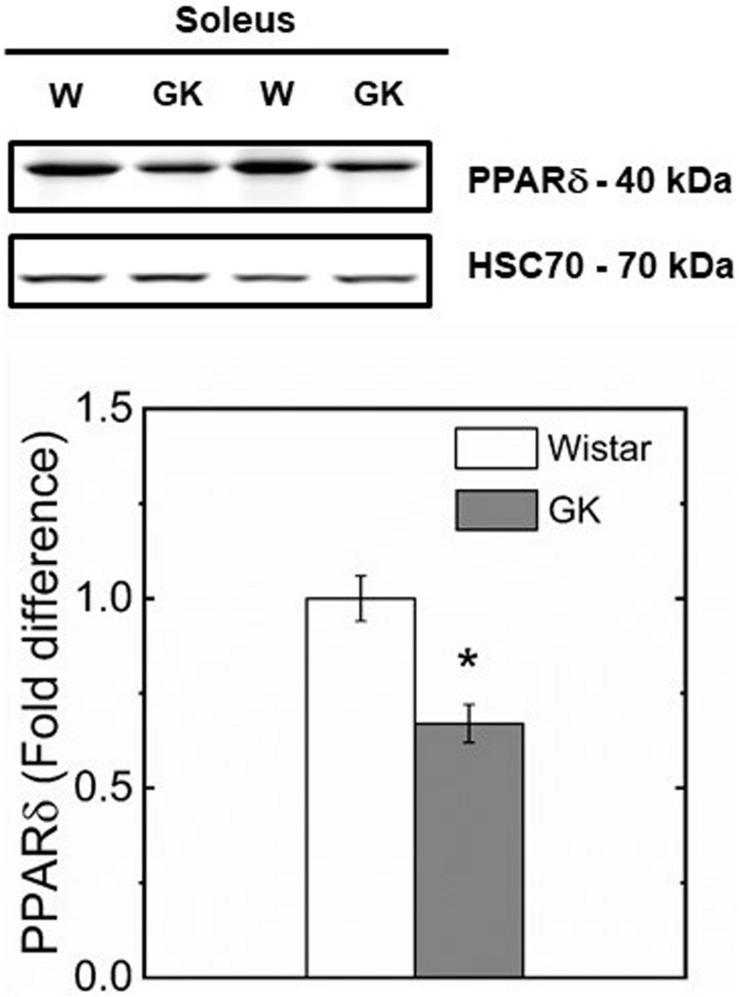
Representative western blots and densitometric analysis of PPARδ protein expression in soleus of control and GK rats. (*) Statistically different from control (*p* < 0.01). Data are mean ± SEM (*n* = 6).

**FIGURE 4:**
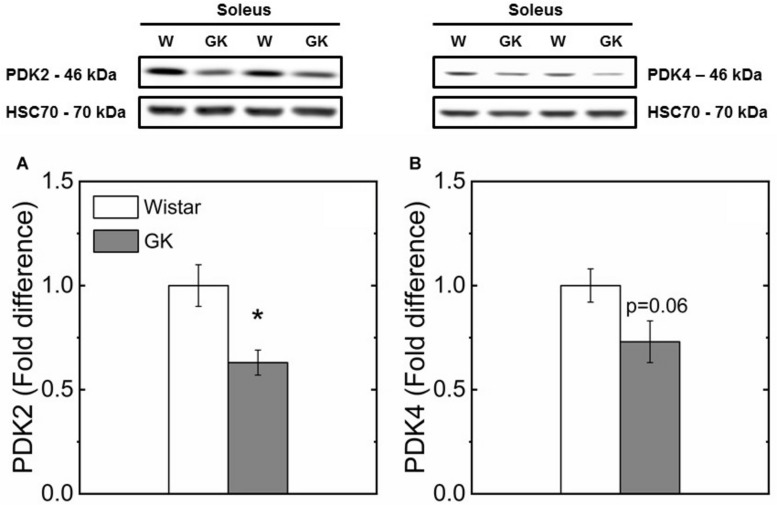
Representative western blots and densitometric analysis of PDK2 **(A)** and PDK4 **(B)** protein expression in soleus of control and GK rats. (*) Statistically different from control (*p* < 0.01). Data are mean ± SEM (*n* = 6).

**FIGURE 5:**
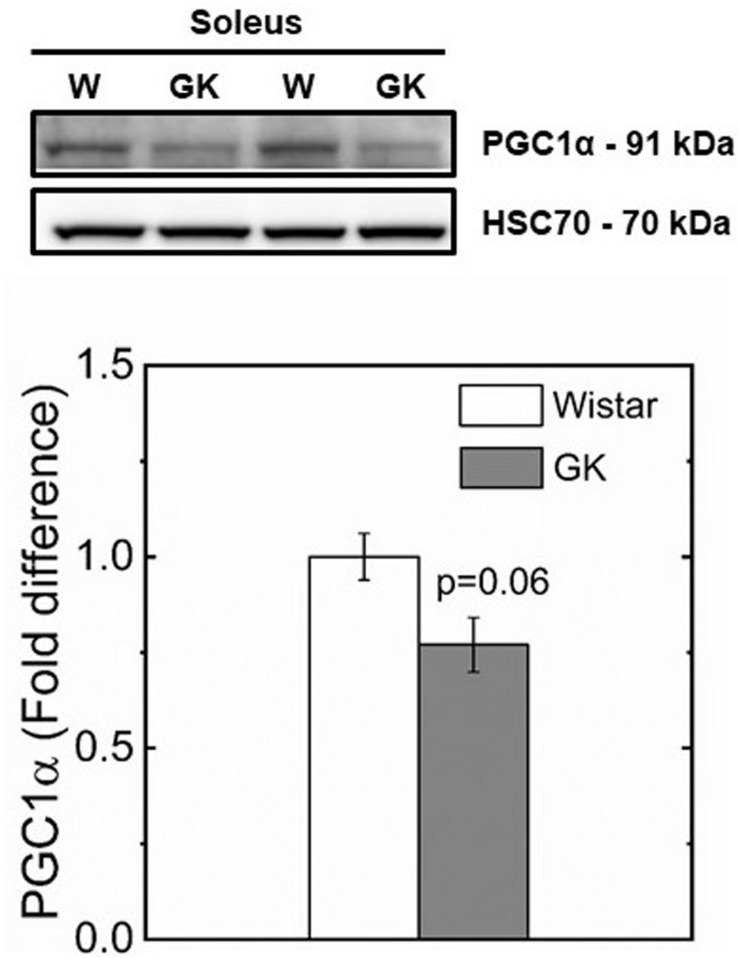
Representative western blots and densitometric analysis of PGC1-α protein expression in soleus of control and GK rats (*p* < 0.06). Data are mean ± SEM (*n* = 6).

**FIGURE 6:**
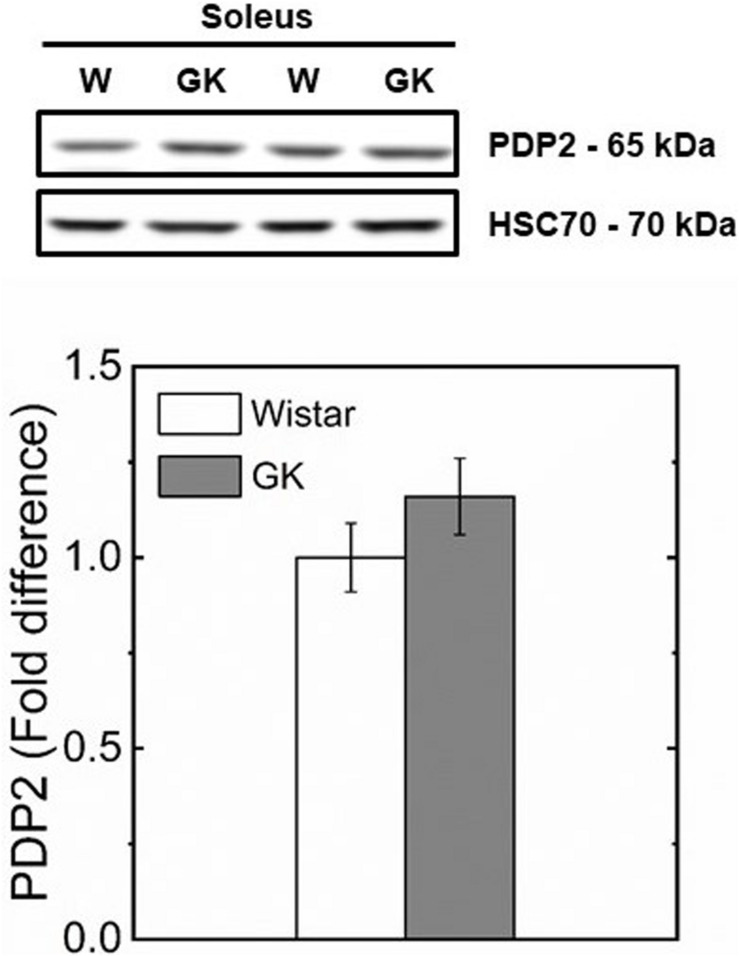
Representative western blots and densitometric analysis of PDP 2 protein expression in soleus of control and GK rats. Data are mean ± SEM (*n* = 6).

Western blot analysis revealed muscle specific differences in protein expression between GK and Wistar. Thus, PPARδ (Figure 3), and PDK2 (Figure 4A) (both *p* < 0.01), and PDK4 (Figure 4B) and PGC1-α (Figure 5, both *p* = 0.06), were reduced in the diabetic group (pyruvate dehyrogenase phosphatase). PDP2 (Figure 6) also was not different between control and diabetic rats.

## Discussion

The GK rat represents a spontaneous non-obese model of T2DM, with insulin-resistance primarily manifesting in skeletal muscle, and was used to evaluate competing fuel selection in the absence of lipid accumulation in oxidative and glycolytic skeletal muscle fibers. Mitochondrial content in both muscle groups was significantly less in GK than in control muscles, whereas similar respiration rates with complex I, II, IV, and fatty acid substrates for both S and WG muscle fibers indicate that mitochondrial function is preserved in the presence of insulin resistance. Substrate competition was observed in both red and white fiber types in control and insulin-resistant skeletal muscle. In particular, the respiration rate in both muscle fiber types in the presence of pyruvate and palmitoylcarnitine was reduced in comparison to pyruvate alone and was further reduced in GK rats. Thus, fuel competition at the level of mitochondrial pyruvate utilization is more impaired in GK than lean non-diabetic control rats even in the presence of reduced PPARδ content, which was hypothesized to enhance pyruvate oxidation.

The role of mitochondria in the etiology of skeletal muscle insulin resistance has been the subject of considerable debate for several decades. In the current study, the finding that mitochondrial function was similar in both diabetic and non-diabetic muscle, despite a reduction in mitochondrial content, adds to the growing body of literature (Boushel et al., 2007; Holloway, 2009; Hoeks et al., 2010; Holloszy, 2013; Muoio, 2014) that contradicts the notion of an intrinsic defect in mitochondrial capacity leading to skeletal muscle insulin resistance and T2DM (Goodpaster, 2013). Thus, alternative mechanisms are required to explain the contribution of the mitochondrion to skeletal muscle insulin resistance. The presence of metabolic inflexibility in individuals with insulin resistance and T2DM suggests that mitochondrial substrate selection may be defective in individuals with T2DM, however, traditional models of insulin resistance that include obesity associated nutrient oversupply add a confounding factor to assessment of this characteristic. We therefore used a non-obese model of insulin resistance to study the flexibility of mitochondrial metabolic fuel competition.

### Mitochondrial Content

Based on CS and SDH activity, mitochondrial content was lower in glycolytic muscle fiber types compared to oxidative, for both groups. This difference is consistent with previous observations indicating a difference in fiber type distributions in these rat muscle groups (Delp and Duan, 1996; Yasuda et al., 2002). Because CS activity is positively related to type IIA (red; fast oxidative and glycolytic) fibers, and inversely related to type IIB (white; fast glycolytic) fiber content (Delp and Duan, 1996), a reduced CS activity in S and WG fibers reported in our study (Table 2) is consistent with the decrease in oxidative fibers and an increase in glycolytic fibers previously reported (Yasuda et al., 2002) in GK rats. In studies of human skeletal muscles, oxidative enzyme activity (He et al., 2001; Oberbach et al., 2006) and type I fiber number are reduced in patients with obesity (Hickey et al., 1995; Tanner et al., 2002; Oberbach et al., 2006) and T2DM (Hickey et al., 1995; He et al., 2001; Oberbach et al., 2006).

The reduced CS and SDH activities observed in GK rats is consistent with a switch in fiber type to lower oxidative capacity triggered by a reduced PGC1-α and PPARδ protein content in muscle observed in our study (Figures 3, 5). In support of this mechanism, a study in which PPARδ was ablated in mouse skeletal muscle (Schuler et al., 2006) showed a key role of PPARδ in regulating type I fiber content via PGC1-α, whereas in human skeletal muscle, PGC1-α and PPARδ mRNA expression were both positively correlated with type I fiber content (Krämer et al., 2007). In our study, the reduced mitochondrial content and preserved function in GK rats, is consistent with the intimate interplay between PPARδ and PGC1-α. Specifically, mitochondrial biogenesis can be triggered by PPARδ activation with the expression of PGC1-α at transcriptional level without affecting mitochondrial function (Ehrenborg and Krook, 2009). These effects did not increase mitochondrial ability to oxidize pyruvate in both control and diabetic groups.

### Mitochondrial Function

The unaltered bioenergetic function evaluated in oxidative and glycolytic muscle is consistent with our previous work on mitochondria isolated from quadriceps muscle (Lai et al., 2017) and on ischemia−reperfusion of skeletal muscle (Liu et al., 2016) of the same GK rats. These studies and a NMR work on this T2DM model suggested the absence of mitochondrial dysfunction in GK rats (Macia et al., 2015). In our parallel GK study (Lai et al., 2017), no significant differences were observed in subsarcolemmal and interfibrillar mitochondrial function evaluated in presence of complex I, II, III, and IV, and fatty acid substrates (palmitoyl-CoA and PCN). In addition, unaltered enzymatic activity of Complex I, II, III, and IV in the insulin-resistant GK muscle mitochondria (Lai et al., 2017) indicated a preservation of biochemical properties of the electron transport chain. Another study on isolated mitochondria from GK skeletal muscle confirmed the absence of mitochondrial bioenergetic dysfunction in this animal model (Lewis et al., 2019) regardless mitochondrial preparations. Thus, both respiring isolated mitochondria and fiber studies confirmed that mitochondrial function is unaltered in insulin-resistant muscles of non-obese GK rats and in T2DM patients (Boushel et al., 2007) when respiration rate are related to citrate synthase (CS) activity used as a mitochondrial marker content.

In our study, there is a trend for higher respiration rates normalized to CS or SDH activity in GK than control (Supplementary Figures 1, 2) for both muscle fibers, but the difference was significant only for respiration rates normalized to SDH (Supplementary Figure 2A, *p* < 0.03). It is possible that the sample size was not sufficient to detect the difference between GK and control. This difference is mainly related to the lower CS and SDH activity in GK than W control. Similarly, to our findings, a previous study indicated that SDH activity in T2DM patients was 25% lower than control subjects in both oxidative and glycolytic fibers (He et al., 2001). Another study on myotubes from T2D subjects attributed the decrease in CS to the absence of a stimulatory insulin effect on CS activity rather than a decrease in mitochondrial content (Ørtenblad et al., 2005). Insulin infusion has been reported to increase CS activity by 28% in human vastus lateralis (Stump et al., 2003).

Mitochondrial function in the permeabilized fiber was preserved not only when stimulated with P, but also in the presence of P and PCN (Figures 1, 2). Thus, the PCN concentration used in our study does not appear to affect the integrity of mitochondrial membrane. In other bioenergetic studies on permeabilized skeletal muscle fibers using the same respiration media (MiR05), the concentration of PCN (75 mM) (Cavalcanti-De-Albuquerque et al., 2014; Lopes Martins et al., 2018) and palmitoyl-CoA (50 mM) (Miotto et al., 2018) were similar to that of our study (60 mM). Also, in another mitochondrial study with a respiration media with 0.25% of BSA (Hansford, 1977), but different from MiR05, PCN concentration was 50 mM. This range of concentration was higher than that used in a study of skeletal muscle mitochondria (Abdul-Ghani et al., 2008). The difference is related, at least in part, to the presence of bovine serum albumin (BSA, 0.1%) in the respiration media. The ability of BSA in binding PCN reduces its free concentration available for mitochondria. Thus, the inhibitory effect of PCN (10 mM) on mitochondrial respiration was removed in presence of a media with 0.1% BSA whereas, in absence of BSA, detergent effects with a decrease in mitochondrial inner membrane potential was reported for a PCN concentration range of 10–50 mM. Also, in plant the PCN-dependent stimulation of mitochondria was shifted to higher concentration (> 50 mM) in presence of BSA greater than 0.1% (Gerhardt et al., 1995).

### Substrate Competition

In our study, substrate competition experiments showed that the capacity of both muscle fiber types to metabolize pyruvate in the presence of PCN was dramatically reduced in GK and controls, but the effect of PCN on pyruvate oxidation was greater in GK than that in control fibers (Figure 2). Our finding agrees with observations reported by other animal and human skeletal (Abdul-Ghani et al., 2008; Kuzmiak-Glancy and Willis, 2014) and heart (Makrecka et al., 2014) studies. In particular, it was reported that the presence of PCN decreases pyruvate maximal respiration rate by 20% and pyruvate utilization by 60% in mitochondria isolated from rat skeletal muscle (Kuzmiak-Glancy and Willis, 2014). Similar results also were reported in another bioenergetic study on mitochondria isolated from mouse and human skeletal muscle (Abdul-Ghani et al., 2008). Although our bioenergetic assays do not quantify the contribution of substrate oxidation to the mitochondrial respiration rate determined in the presence of competing fuels, they suggest that the greater respiration rate observed with pyruvate alone than that with PCN and pyruvate is related to an inhibitory effect on mitochondrial utilization of pyruvate. In support of this view, it should be considered that the reduced rate of mitochondrial respiration stimulated by competing complex I substrate and free fatty acid metabolites should not be attributed to the CI capacity to transport electron.

To determine whether the difference between GK and control on the inhibitory effect of PCN on the respiration rate in the presence of P was related to the oxidative enzyme activity (i.e., CS and SDH) or muscle fiber mass, we calculated the changes in respiration rate PCN_P_ and PCN + P_P_ relative to that obtained with P_P_ because these changes are independent of the muscle fiber mass or CS content. Even in this case, the decrease of the respiration rate PCN + P_P_ (57–63%) in GK fibers was significantly greater than that in control (37–25%) muscle fibers (Figures 2C,D) indicating that PCN effect on respiration differs between GK and control rats. Taken together, this evidence suggests that PCN outcompetes pyruvate for mitochondrial respiration in skeletal muscle of GK rats to a greater extent. Thus, this effect could contribute to the impairment of glucose utilization in insulin resistant skeletal muscle whenever fuel competition occurs at a mitochondrial level even in the absence of obesity.

Whole body fuel utilization data have been reported for the GK model in a study on glucose telemetry analysis with indirect calorimetry measurements (Iuchi et al., 2017). In this study, for both dark and light periods, GK rats had similar respiratory exchange ratios as the control group. Thus, this study suggests an unaltered whole-body substrate utilization whereas our results indicate a mitochondrial disposition to prefer fatty acids in this non-obese animal model of T2DM. Nevertheless, this comparison is limited by the experimental design of whole-body measurements, which does not distinguish between fed and fasting states, whereas mitochondrial respiration rates with competing substrates are mimicking the transition from fasting to the fed state. Substrate preference differences between mitochondrial and whole-body may be due to tissue specific differences in substrate selection that cannot be detected with indirect calorimetry (Petersen et al., 2015). Our data on non-obese rats with T2DM are consistent with the inability of mitochondria to switch from lipid to glucose utilization observed in lean insulin-resistant patients (Petersen et al., 2015) and suggest that studies are warranted on this relationship.

In this study, we investigated whether PPARδ as key regulator of skeletal muscle substrate selection (Wang et al., 2003; De Lange et al., 2008) in T2DM, had an effect on mitochondrial fuel competition between PCN and pyruvate. A reduced content of PPARδ protein in GK mirrors a reduced PDK4 (Figure 4B) which is responsible for PDH phosphorylation and an unaltered content of PDP2 and PDP4 which catalyzes PDH dephosphorylation to restore its activity. Under this condition, PDH is expected to be more active for pyruvate oxidation in GK than control because the content of PDK4 responsible for inactivation of PDH is reduced in GK in comparison to control, while PDP2/4 content is similar in both animal groups (Figure 6). In contrast to this view, our results showed that pyruvate oxidation in GK fibers is not enhanced with M + P substrate. The respiration rate in the presence of pyruvate is due to the NADH availability depending on transport of pyruvate into mitochondria and conversion of pyruvate to Acetyl-CoA by PDH. Although PDH activity was not determined, we can infer that PDH activity was enough to sustain pyruvate utilization for both mitochondria of GK and control with no impairment for GK rats because the respiration rate with pyruvate was similar in both GK and W rats. If PDH activity is limiting the respiration rate with pyruvate, PDH activity would be similar in both GK and W rats. And if PDH activity is not limiting the bioenergetics, PDH activity difference between GK and W rats may exist. Thus, our study suggests that mitochondrial pyruvate utilization and fuel competition in the presence of PCN was not affected by reduced PPARδ.

The glucose-fatty acid cycle proposed by Randle (Randle et al., 1988; Randle, 1994) could in part explain the effect of PCN on mitochondrial pyruvate oxidation. In particular, oxidation of fatty acids and carbohydrate generates a high concentration of acetyl-CoA, which is an allosteric inhibitor of PDH (Randle et al., 1988; Randle, 1994). However, this mechanism cannot explain the inhibitory effect of palmitoyl-CoA and oleoyl-CoA on glutamate (Kuzmiak-Glancy and Willis, 2014) and succinate (Abdul-Ghani et al., 2008) oxidation in mitochondria, because it does not involve PDH activity. Thus, these studies support our interpretation of an inhibitory effect of PCN on pyruvate oxidation without PDH involvement (Liepinsh et al., 2017). To further probe for competing fuel utilization, different combinations and orders of substrates can be used. In addition to using complex I and II substrates, glyceraldehyde-3-phosphate is of particular interest because it would provide insight into the interplay between mitochondrial glycerol-3-phosphate dehydrogenase, b-oxidation and glycolysis (Mráèek et al., 2013).

The interpretation of the effect of PCN on mitochondrial fuel utilization in the context of the regulation of glucose and fat oxidation pathways in fasting and fed state and during exercise should be made with caution. Muscle alternates the utilization of glucose and fat oxidation in fasting and fed states. In a postprandial state, the switch from fat to carbohydrate utilization occurs by restricting the availability of substrates for b-oxidation (McGarry, 2002). This is caused by malonyl-CoA inhibiting carnitine palmitoyltransferase-1 (CPT-1), which is responsible for the conversion of long-chain fatty acyl-CoAs to acylcarnitines (e.g., PCN) (Kerner and Hoppel, 2000; Muoio, 2014). In our study, PCN access to mitochondria is not limited by CPT-1 since it is transported via carnitine-acylcarnitine translocase. Thus, the effects of PCN excess in diminishing mitochondrial utilization of pyruvate may be attenuated *in vivo* when the flow of long-chain fatty acid is more realistically controlled by CPT-1. Nevertheless, a bioenergetic study on healthy skeletal muscle reported that mitochondrial utilization of pyruvate was inhibited not only by PCN, but also by palmitoyl-CoA, which is a CPT-1 dependent substrate (Abdul-Ghani et al., 2008). Consistent with this observation and our findings, fatty acids have been reported to inhibit glycolysis not only in skeletal muscle (Jenkins et al., 1988), but also in heart (Cortassa et al., 2020).

Furthermore, the conditions used in our fuel utilization experiments presented some similarities with those during exercise. In particular, the high ADP and pyruvate concentration used in our work may have contributed to PDH activation as in exercise (Spriet and Heigenhauser, 2002; Lundsgaard et al., 2018) because PDK, an enzyme that inactivates PDH complex, is inhibited in presence of pyruvate and ADP (Pratt and Roche, 1979; Mann et al., 2000). Thus, in our study, the experimental conditions of the permeabilized respiring fibers are unlikely to resemble resting physiological conditions in which PDH is not active like in presence of high ADP concentrations. The bioenergetic approach proposed does not allow to directly relate the findings on mitochondrial utilization of competing substrates to the fuel utilization at resting conditions. Nevertheless, it is reasonable to assume that the effect of PDH activation due to ADP was similar for both respiration rates obtained with P or PCN + P_P_ since the concentration of P and ADP was the same in both experiments.

Other mechanisms involving the electron transport chain and membrane potential rather than PDH and PDK are proposed to explain alterations in mitochondrial metabolic flexibility (Abdul-Ghani et al., 2008; Sahlin et al., 2008; Hue and Taegtmeyer, 2009). It has been suggested that fatty acids may impair the electron transport chain in complex I and III (Galgani et al., 2008). The inhibition of NADH oxidation may be caused by a specific interaction of long chain fatty acids with complex I rather than a detergent-related effect of palmitoyl-CoA and palmitoyl carnitine (Batayneh et al., 1986). Another potential substrate competition mechanism is related to the level of mitochondrial uncoupling induced by fatty acids. It was reported that a decrease in membrane potential would inhibit metabolite transport in mitochondria in favor of fat oxidation (Hue and Taegtmeyer, 2009). Nevertheless, the mechanisms proposed do not explain a more pronounced effect of PCN on pyruvate oxidation rate in GK than that observed in control for both muscle groups.

Proteins of the sirtuin family have shown to have a key role in substrate utilization in skeletal muscle (Jing et al., 2013; Lantier et al., 2015). A reduced SIRT3 content has been shown to increase insulin resistance (Lantier et al., 2015) and impair glucose oxidation by inhibition of PDH in favor of fatty acid utilization in skeletal muscle (Jing et al., 2013). Thus, mitochondrial utilization of competing substrates observed for the insulin resistant GK rats may have been affected by SIRT3. Nevertheless, as previously discussed, indirect evidence suggests the absence of difference in PDH activity effects on bioenergetics between GK and W rats.

In our study, we used S and WG muscle fibers as representative of oxidative and glycolytic muscle fibers, respectively. For both muscle fibers, mitochondrial fuel competition was greater in insulin resistant muscle of GK rats in comparison to the control group. This result is relevant in determining the mechanisms responsible for muscle fuel utilization in fed and fasting states. In particular, it has been suggested that type I and II fibers contribute differently to glucose utilization leading to a different effect on insulin resistance. Glucose uptake was found to be linearly related to type I fiber and inversely related to type II fibers in healthy patients (Lillioja et al., 1987). Some studies suggest a primary role of oxidative muscle fibers in insulin resistance, while others reported a similar insulin resistance in different muscle fibers although different muscle fibers appear to have similar sensitivity for phosphoregulation by insulin (Albers et al., 2015). In our study, long chain acylcarnitine can alter substrate utilization in mitochondria of both muscle fiber types. Thus, a common mechanism could be responsible for the fuel competition observed in skeletal muscle. The role of long chain acylcarnitine is important in the development of metabolic disorders and our study underscores the need for further research aimed at understanding the specific mechanisms by which long chain acylcarnitines impair metabolic flexibility in both insulin resistant muscle fiber types.

### PPAR δ/β and Fuel Utilization

In this study, we investigated whether PPAR δ/β had an effect on mitochondrial fuel competition between PCN and P. PPAR δ/β, a key regulator of skeletal muscle substrate selection (Wang et al., 2003; De Lange et al., 2008), is a promising target for treatment of obesity, dyslipidemia, T2D and non-alcoholic fatty liver disease (Luquet et al., 2005; Palomer et al., 2018). Human (Krämer et al., 2007; Risérus et al., 2008) and animal (Brunmair et al., 2006; Constantin et al., 2007) studies suggest that PPAR δ/β may rescue impaired fatty acid utilization in T2D due to its effects on substrate selection signaling pathways. In particular, metabolic flexibility may be mediated by overexpression of PDK enzymes (Randle, 1994; Zhang et al., 2014) by their ability to regulate PDH activity, which has a key role in glucose oxidation, and by overexpression of b-oxidation and CPT1 genes, thus contributing to an increase of fat catabolism (Tanaka et al., 2003; Wang et al., 2003). In our study, the reduced protein expression of PPAR δ/β and PGC1-α in our animal model of T2DM is consistent with the reduced gene expression of PPAR δ/β and PGC1-α that has been reported in patients with T2DM (Mensink et al., 2007) in comparison to control obese. Because skeletal muscle PPAR δ/β increases during fasting (Ehrenborg and Krook, 2009) whereas the animals of our study had access to food, it is possible that the PPAR δ/β effects were diminished.

Furthermore, it has been suggested that PPAR δ/β activation by free fatty acids (Staiger et al., 2009) may be a mechanism to prevent the accumulation of fatty acid (Georgiadi and Kersten, 2012). Thus, in our study, we speculate that the acute mitochondrial stimulation with PCN, triggered an activation of the PPAR δ/β. The difference observed between diabetic and control animals could be related to the target genes of PPAR δ/β such as Forkhead Box O1A (FOXO1). Thus, in the presence of insulin resistance as in GK rats, the impaired ability of insulin to deactivate FOXO1 may have enhanced fatty acid utilization. FOXO1 not only induces PDK4 (Kim et al., 2006; Nakamura et al., 2014), but also membrane enrichment in CD36 with an increase of fatty transport and utilization (Bastie et al., 2005; Nahlé et al., 2008). The transcriptional activity of FOXO1 is activated when insulin concentration is low.

In summary, we found that mitochondrial function is preserved in insulin-resistant skeletal muscle and that there is a significant effect of FFA metabolites on mitochondrial fuel utilization determining a reduced carbohydrate oxidation substrate, even in the presence of reduced PPARδ expression, which would be expected to predispose mitochondria to utilize more carbohydrate. In the absence of obesity, mitochondrial metabolic inflexibility is still present and appears related to a mitochondrial impairment related to long-chain acylcarnitines. The impact of mitochondrial dysfunction on metabolic flexibility in T2DM patients has yet to be determined. New evidence suggests that mitochondria are the primary site controlling fuel selection and a common impairment of mitochondrial utilization of competing fuels is present in oxidative and glycolytic fibers in insulin resistant skeletal muscle of non-obese rats.

## Materials and Methods

### Animals

The GK non-obese rat with type 2 diabetes mellitus (T2DM) and Wistar (Control) control animals were purchased from Charles River Laboratories (Wilmington, MA, United States). GK rats manifest spontaneous skeletal muscle and hepatic insulin resistance, mild hyperglycemia and normal lipidemia by 4 weeks of age. Six male GK and six Wistar controls were housed in pairs in the Animal Resource Center facilities of Case Western Reserve University with 12:12-h light-dark cycle and were fed standard diet chow (Prolab Isopro RMH 3000, LabDiet, St. Louis, MO, United States) *ad libitum*. The sample size was used to detect difference between bioenergetics parameters in rat skeletal muscle fibers (Lopes Martins et al., 2018). Male animals were selected to avoid any hormonal effects on energy metabolism during the menstrual cycle. Animals were euthanized at 28 weeks of age. The euthanasia was performed 7–8 a.m. on the day of study while food was available. All procedures were approved by Case Western Reserve University Institutional Animal Care and Use Committee and performed in accordance with the National Research Council guidelines for care and use of laboratory animals in research. Plasma insulin and glucose, and the bioenergetics of skeletal muscle mitochondria in these animals were previously published by our group (Lai et al., 2017).

### Buffers

All reagents were purchased from Sigma, unless otherwise specified. Mitochondrial respiration medium (MiR05), relaxing and biopsy preservation solution (BIOPS) and saponin solution for muscle permeabilization were prepared as described previously (Veksler et al., 1987; Pesta and Gnaiger, 2012). MiR05 consists of 0.5 mM EGTA, 3 mM MgCl_2_.6H_2_O, 60 mM K-lactobionate, 20 mM Taurine, 10 mM KH_2_PO_4_, 20 mM HEPES, 110 mM D-Sucrose, 1 g/l BSA, essentially fatty acid free. The pH of MiR05 was adjusted to 7.1 with KOH at 30°C. BIOPS consists of 1.77 mM CaK_2_EGTA, 7.23 mM K_2_EGTA, 5.77 mM Na_2_ATP, 6.56 mM MgCl_2_.6H_2_O, 20 mM Taurine, 15 mM Na_2_Phosphocreatine, 20 mM Imidazole, 0.5 mM DTT, 50 mM MES hydrate. The pH of BIOPS was adjusted to 7.1 with KOH at 0^*o*^C. Saponin stock solution was prepared fresh everyday by dissolving 5 mg Saponin in 1 mL BIOPS.

### Skeletal Muscle Fiber Preparation

Permeabilized skeletal muscle fibers were prepared as described previously (Pesta and Gnaiger, 2012). White gastrocnemius (WG) and soleus (S) muscles were removed and transferred into 10 mL of BIOPS on ice. Connective and fat tissue were removed, the muscle was cut into 50–100 mg fragments, and placed with BIOPS onto an ice-cold petri dish. The fiber bundles were mechanically separated with a pair of sharp forceps over a standardized period of 5 min for a ∼4 mg sample. An optical glass binocular magnifier with a 10× magnification lens was used to verify the degree of fiber separation. A color change from red to pale fibers also was used as a criterion to evaluate the degree of separation of the soleus muscle. The fibers were gently teased apart and stretched out. The bundles of fibers were permeabilized by gentle agitation for 30 min at 4°C in a solution of 50 μg of saponin per mL of BIOPS. Fibers were washed for 10 min by gentle agitation in ice-cold MiR05 and subsequently blotted, weighed, and then immediately used for respirometry measurements.

### High-Resolution Respirometry

Mitochondrial respiration was determined as described previously (Lemieux et al., 2010). Permeabilized fibers (1.0–2.5 mg) were transferred to the chamber of the polarographic system (OROBOROS-O2k, Innsbruck, Austria, Gnaiger, 2008) containing 2 mL of MiR05. Datlab software (OROBOROS Instruments) was used for data acquisition and to calculate oxygen consumption in the fibers. The metabolic chamber temperature was maintained at 37°C.

The protocol (Protocol 1) used to evaluate mitochondrial function with complex I, II, and IV substrates, uncoupler, and inhibitors was performed according to the following sequence: malate (5 mM), pyruvate (5 mM), ADP (2.5 mM), cytochrome *c* (Cyt-c; 10 μM), succinate (10 mM), dinitrophenol (DNP; titration up to an optimum concentration, 5–20 μM, until respiration reaches plateau), rotenone (0.5 μM), antimycin A (2.5 μM), ascorbate (4 mM), tetramethylphenylenediamine (TMPD; 0.5 mM), and azide (100 mM). The amount added to the chamber is referred to as the final concentration. All mitochondrial respiration rates were corrected for oxygen flux to account for instrument background. The difference between the uncoupled mitochondrial respiration rate (ET) and that in the presence of rotenone (R), which quantifies mainly the contribution of C-II to uncoupled oxidation, was used to determine NADH-linked respiration rate (ET-R), while the azide sensitive respiration rate in the presence of ascorbate plus TMPD was used to determine complex IV respiration rate (C-IV). Respiration rate in the presence of antimycin A was subtracted from all mitochondrial respiration rates, and respiratory flux was expressed in pmol of O_2_ s^–1^ mg^–1^ ww of fibers. The respiratory acceptor control ratio (RACR) was computed as the ratio of the oxphos state respiration rate (P_P_) to the leak state respiration rate (P_L,N_) with no adenylates.

Another protocol was used to evaluate mitochondrial fatty acid oxidation and substrate selection in muscle fibers. The substrates were added in sequence: malate (5 mM), palmitoylcarnitine (60 μM) (Cavalcanti-De-Albuquerque et al., 2014; Lopes Martins et al., 2018; Miotto et al., 2018), ADP (2.5 mM), pyruvate (5 mM) and cytochrome c (Cyt-c; 10 μM). The palmitoylcarnitine concentration was determined by titration. The amount added to the chamber is referred to as the final concentration. Respiration rate obtained with palmitoylcarnitine, pyruvate and malate was compared with data obtained in the presence of only pyruvate + malate in protocol 1.

### Citrate Synthase and Succinate Dehydrogenase Activities

Frozen tissue samples were weighed, and tissue homogenates prepared in 5% cholate, 25 mM KPi/2 mM EDTA buffer (pH 7.4) and protease inhibitors to a final concentration of 10 mg/mL using a hand-held glass-on-glass homogenizer. Homogenate was centrifuged for 5 min at 2000 rpm at 4°C in a table-top centrifuge. Citrate synthase (CS) and succinate dehydrogenase (SDH) activities were measured using a spectrophotometer at 412 and 600 nm, respectively (Hoppel et al., 1987).

### Western Blot

Soleus muscle homogenates were prepared by grinding muscle tissue with ice-cold lysis buffer (Invitrogen) in the presence of protease inhibitor cocktail, 5 mM phenylmethylsulfonyl fluoride (Sigma), and Phos-STOP (Roche Applied Sciences, Indianapolis, IN, United States). Samples for Western blot were prepared from supernatants after centrifugation of homogenates for 10 min at 14,000 g. Protein concentrations were measured using a BCA protein assay kit (Pierce Biotechnology, Rockford, IL, United States). A 50 μg of muscle homogenate was solubilized in Laemmli sample buffer containing 5% β-mercaptoethanol and boiled for 5 min. Proteins were separated on 4–20% Novex Tris Glycine SDS-PAGE Electrophoresis (Invitrogen), transferred to a nitrocellulose membrane (0.22 μm pore – LiCor Biosciences), and blocked with 5% bovine serum albumin in tris-buffered saline with 0.1% Tween-20 (TBST) for 1 h. Membranes were then incubated overnight with anti-PPARδ (1:1000 dilution, PA5-29678, Thermo Fisher Scientific, Pittsburgh, PA, United States), anti-PGC1-α (1:1000 dilution, sc-33796, Santa Cruz Biotechnology, Dallas, TX, United States), anti-PDP2 (1:1000 dilution, NBP1-82912, Novus Biologicals, Littleton, CO, United States), anti-PDK2 (1:1000 dilution, Ab68164, Abcam, Cambridge, MA, United States), anti-PDK4 (1:1000 dilution, 12949-1-AP, Proteintech, Chicago, IL, United States), and anti-HSC70 (1:5000 dilution, sc-7298, Santa Cruz Biotechnology, Dallas, TX, United States) antibodies. Membranes were washed in TBST, and with the exception of the PGC1-α membrane (see below), incubated with IRDye^®^ 680RD Goat anti-Mouse IgG- or IRDye^®^ 800CW Goat anti-Rabbit IgG -conjugated secondary antibodies (1:10,000 dilution, LiCor Biosciences, Lincoln, NE, United States) for 2 h. After three 5 min washes in TBST, antibody binding was detected using an Odyssey CLx (LiCor Biosciences, Lincoln, NE, United States) and quantified using Image Studio software (LiCor Biosciences, Lincoln, NE, United States). Detection of PGC1-α and its associated loading control was detected by chemiluminescence using the ECL prime reagent (GE Healthcare, Chicago, IL, United States) and quantified using ImageJ software (NIH, Bethesda, MD, United States).

### Statistical Analysis

The results are reported as means ± SEM. The comparison of activity of the enzyme markers for mitochondrial content between control and diabetic rats was analyzed using a two tailed Student *t*-test and two-sample equal variance. Also, the comparisons of respiration rates obtained with the protocol to evaluate the function of the electron transport chain components, were analyzed with Student *t*-test. Difference of *p* < 0.05 was considered significant. The comparisons of the respiration rates obtained with competing substrates between control and diabetic rats were evaluated with two-way ANOVA with Bonferroni-Holm correction for multiple comparisons.

## Data Availability Statement

The datasets generated for this study are available on request to the corresponding author.

## Ethics Statement

All procedures were approved by the Case Western Reserve University Institutional Animal Care and Use Committee and performed in accordance with the National Research Council guidelines for care and use of laboratory animals in research.

## Author Contributions

NL and CF contributed to the conception and design of the study, performed the statistical analysis, and wrote the first draft of the manuscript. NL, CF, CK, and SC performed the experiments. NL, CF, CK, SC, CH, and JK analyzed the data. NL, CH, and JK provided the resources. All authors contributed to manuscript revision, read and approved the submitted version.

## Conflict of Interest

The authors declare that the research was conducted in the absence of any commercial or financial relationships that could be construed as a potential conflict of interest.
