# Supplementary material for: Mitochondrial Utilization of Competing Fuels Is Altered in Insulin Resistant Skeletal Muscle of Non-obese Rats (Goto-Kakizaki)
Source: Front Physiol. 2020 Jun 16;11:677. doi: 10.3389/fphys.2020.00677 (PMC7308651; doi:10.3389/fphys.2020.00677)
Supplement: Supplementary file 1 [file Presentation_1.pdf]

Supplementary Material

Supplementary Figures

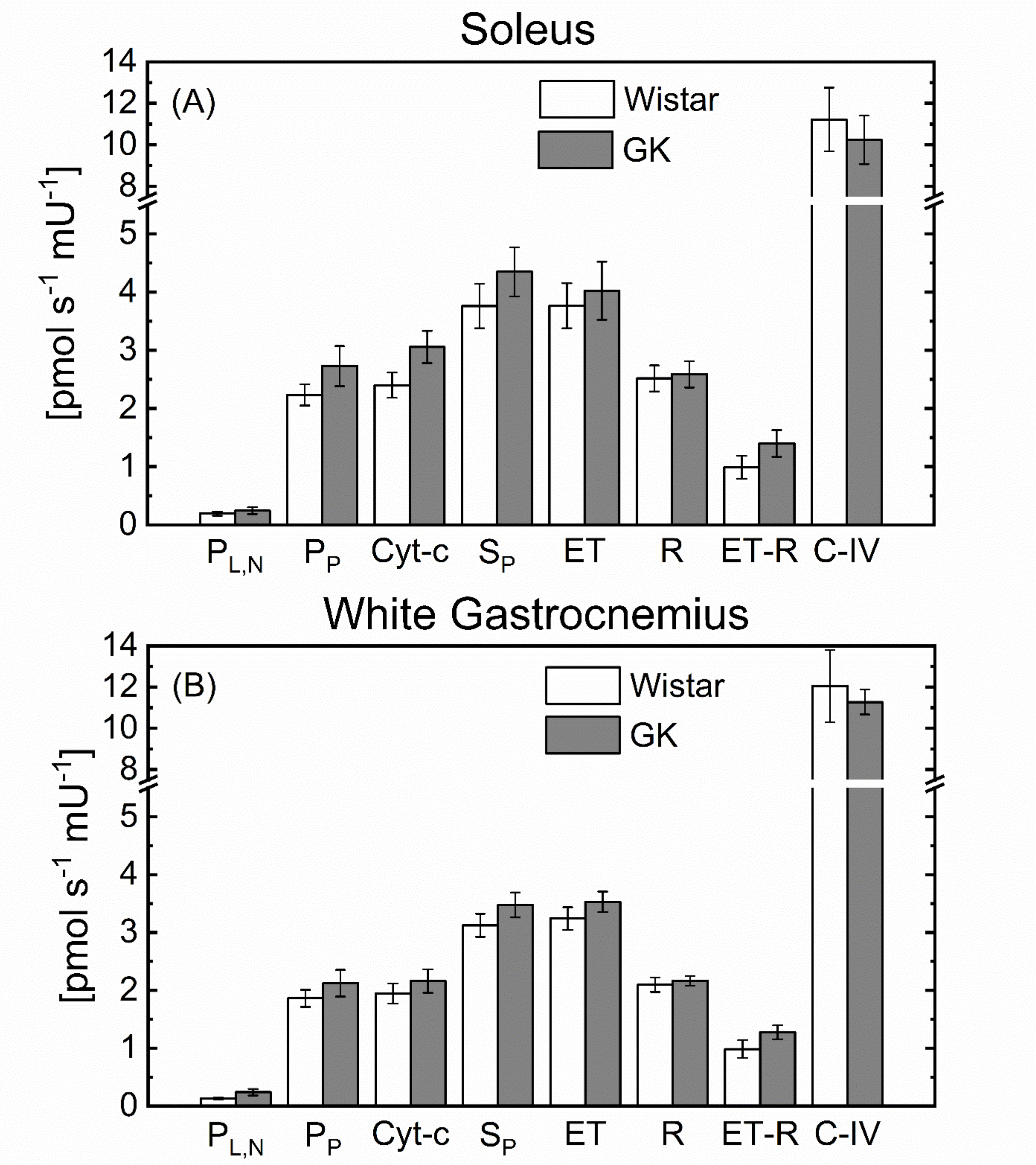

Supplementary Figure 1. Respiration rates normalized to citrate synthase activity in permeabilized soleus (**A**) and white gastrocnemius (**B**) of control and GK rats obtained under leak ($P_{L,N}$) and oxphos state (P_P) with malate and pyruvate, cytochrome c (Cyt-c), Succinate (S_P), uncoupled mitochondria to measure maximal electron transport chain capacity (ET), rotenone (R). Also, ET-R indicates the respiration rate difference between that indicated with ET and R; CIV indicates the respiration rate difference between that with ascorbate plus TMPD and azide. Data are mean ± SEM (n=6).

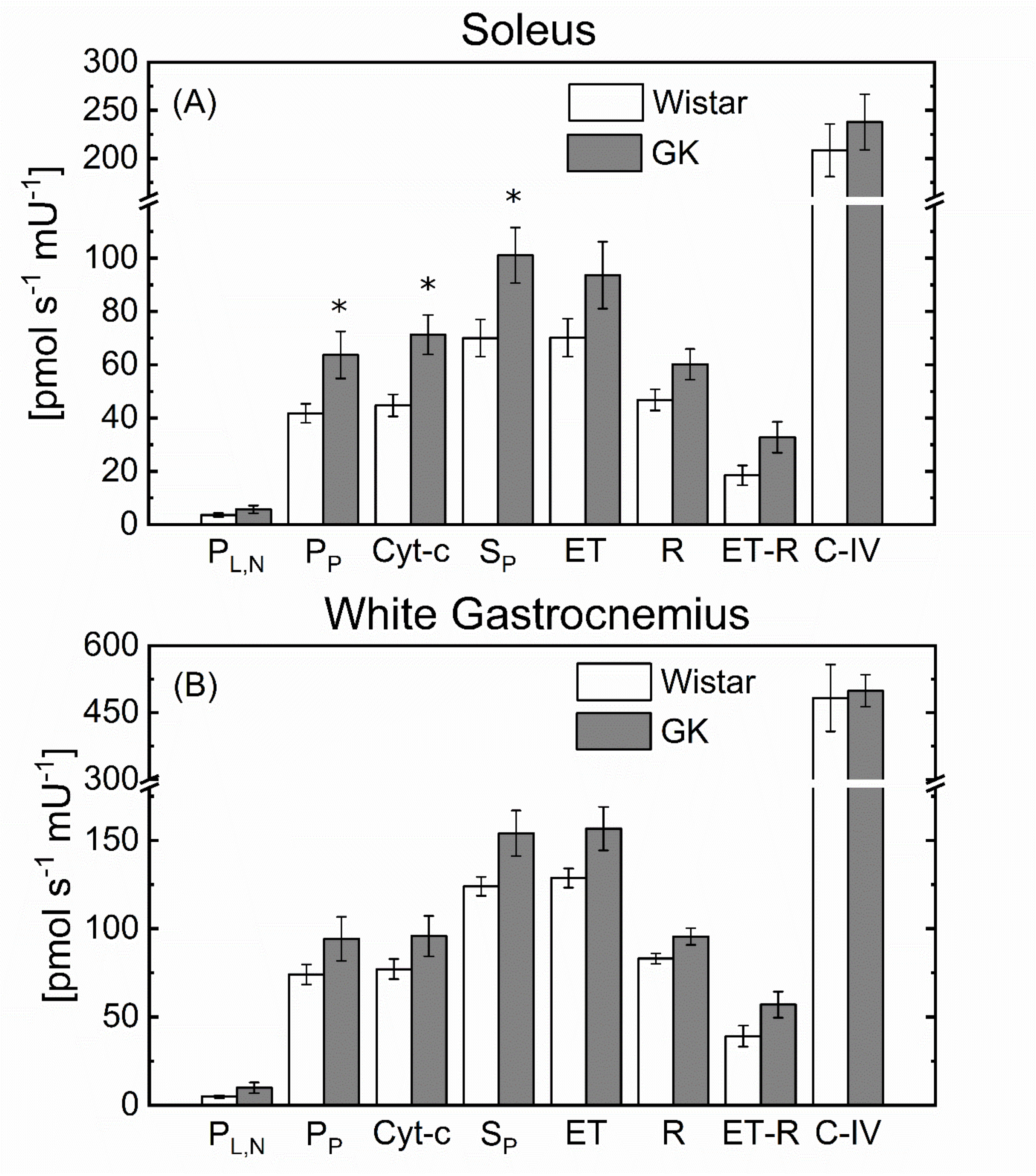

Supplementary Figure 2. Respiration rates normalized to succinate dehydrogenase activity in permeabilized soleus (**A**) and white gastrocnemius (**B**) muscles of control and GK rats obtained under leak state ($P_{L,N}$) and oxphos state (P_P) with malate and pyruvate, cytochrome c (Cyt-c), Succinate (S_P), uncoupled mitochondria to measure maximal electron transport chain capacity (ET), rotenone (R). Also, ET-R indicates the respiration rate difference between that indicated with ET and R; CIV indicates the respiration rate difference between that with ascorbate plus TMPD and azide. (*) Statistically different from control. Data are mean ± SEM (n=6).
